# Effects of esketamine and fluoxetine on depression-like behaviors in chronic variable stress: a role of plasma inflammatory factors

**DOI:** 10.3389/fpsyt.2024.1388946

**Published:** 2024-05-15

**Authors:** Haixia Chen, Xinxin Zhao, Xinxu Ma, Hongzhe Ma, Cuihong Zhou, Yunyun Zhang, Zhengwu Peng, Shanshan Xue, Min Cai

**Affiliations:** ^1^ The College of Life Sciences and Medicine, Northwest University, Xi’an, China; ^2^ Department of Anesthesiology and Perioperative Medicine, Xijing Hospital, The Fourth Military Medical University, Xi’an, Shaanxi, China; ^3^ Department of Psychiatry, Xijing Hospital, The Fourth Military Medical University, Xi’an, Shaanxi, China

**Keywords:** esketamine, fluoxetine, major depressive disorder, chronic variable stress, inflammatory cytokines

## Abstract

Mounting evidence has identified the rapid and sustained antidepressive and anxiolytic-like effects of esketamine. However, the underlying mechanism of this no-monoamine target rapid-onset antidepressant is still underexplored. Immune-inflammatory pathways and cell-mediated immune activation, mainly including inflammatory cytokines in plasma, play a pivotal role in the pathogenesis of major depressive disorder and are also a potential therapeutic target for MDD. The current study was designed to clarify the role of esketamine on the expression of plasma cytokines in a depressive-like model introduced by chronic variable stress (CVS). In this study, a 21-day consecutive CVS protocol was applied to produce depressive- and anxiety-like behaviors. After the single dose or 7-day repeated administration of esketamine or fluoxetine, the depressive- and anxiety-like behaviors and the expression of inflammatory cytokines in plasma were examined. Both a single dose of esketamine and 7-days repeated fluoxetine administration elicited anti-depressive and anxiolytic effects in mice exposed to CVS. Additionally, CVS produced significant changes in the plasma inflammatory factors, notably increasing the expression of IL-1β, IL-6, IL-8, IL-17A, TNFα, IL-4, IL-9, IL-24, IL-37, IFN-β, and CXCL12, while reducing IL-10 and IL-33. With the administration of esketamine and fluoxetine, CVS-produced inflammatory disturbances were partially normalized. Together, our findings provide a novel insight that acute esketamine treatment could rescue CVS-produced depressive-like and anxiety-like behaviors in mice by normalizing the expression of inflammatory cytokines; this effect was similar to the repeated administration of fluoxetine. These results contributed to the understating of rapid anti-depressant effects elicited by esketamine.

## 1 Introduction

Major depressive disorder (MDD) is a highly prevalent and debilitating mental disorder worldwide with substantial morbidity and mortality, often compounded by symptoms of anxiety or even comorbidity with anxiety disorders (1). The pandemic of COVID-19 further exacerbated MDD incidence by 27.6%, significantly contributing to the global burden of the disease (2). For the past half-century, pharmacotherapy against MDD has predominantly relied on monoaminergic anti-depressants, including selective serotonin reuptake inhibitors (SSRIs) or serotonin and norepinephrine reuptake inhibitors (SNRIs), rooted in the monoamine transmitter hypothesis. These pharmaco-therapeutics exhibit limitations in clinical efficacy, including delayed onset of response, adverse side effects, and inadequate response rates ranging from 30% to 50% among MDD patients (3–6). Therefore, there is an urgent need to develop novel medication candidates for the treatment of MDD in the future.

R, S-ketamine emerges as a promising non-monoamine anti-depressant for MDD, demonstrating remarkably rapid and robust anti-depressant effects in MDD patients and depressive-like rodents (6). In 2019, the S-enantiomer of ketamine, esketamine (Spravato), was approved in Europe as a treatment option for medication-resistant depression in combination with SSRIs/SNRIs (7). Subsequently, this agent was also approved by the U.S. FDA as a new rapid-acting anti-depressant for patients with medication-resistant depression or suicide ideation, owing to its capability of alleviating symptoms within hours (8). Recent years have witnessed a surge in studies (9) investigating the underlying mechanism of the esketamine’s rapid onset anti-depressive potency. Primarily, esketamine exerts its anti-depressant effect by NMDA receptor inhibition and membrane stabilization via the modulation of intracellular ionic flow of Na+ and Ca2+ (10, 11) Additionally, recent studies have reported that the esketamine’s rapid anti-depressant action involves upregulation of brain-derived neurotrophic factor (BDNF) mediated by sirtuin type 1 activation (SIRT1) and rapamycin complex target (mTORC) signaling pathway (12–15). However, the precise mechanisms of the rapid anti-depressant effects of esketamine remain incompletely understood.

Accumulating evidence suggested that abnormalities in immune-inflammatory pathways and activation in the cell-mediated immune system play pivotal roles in the pathophysiology of MDD (16). Chronic stress induced the upregulation of inflammatory activity and the interaction of biological mediators of inflammatory responses between peripheral and central nervous system to elicit neurological and depressive-like behavior changes. Clinical studies have consistently demonstrated the elevated plasma inflammatory response in patients with MDD, characterized by increased expression of granulocyte and monocyte, up-regulated levels of acute-phase reagents including c-reactive protein (CRP) and haptoglobin (17, 18), inflammatory cytokines (19, 20), and possibly chemokines (21), compared with healthy controls. Notably, these effects are partly attributed to reducing pro-inflammatory cytokines and inhibiting microglial activation (22, 23). Moreover, the normalization of over-responded plasma inflammatory cytokines has been shown to correlate with improved depressive symptoms in MDD patients. Overall, alterations in peripheral inflammatory mediators may constitute an underlying mechanism of antidepressants. However, whether the rapid-onset or delayed-onset antidepressants, for instance, esketamine or ketamine, equally affected the plasma inflammatory cytokines still necessitates further investigation.

The current study aimed to clarify the changes in plasma inflammatory cytokines in mice treated with an *i.p.* injection of a single dose of esketamine or 7-day repeated fluoxetine after 3 weeks of chronic variable stress (CVS) exposure.

## 2 Materials and methods

### 2.1 Animals

Male C57BL/6J male mice (6-8 weeks of age, weighing 18-22 g) were obtained from the Animal Center of the Fourth Military Medical University (Xi’an, China). All experimental procedures were applied by the Institutional Animal Care and Use Committee of the Fourth Military Medical University. Mice were housed at constant room temperature with a 12-hour light/dark cycle (light duration was 08:00 to 20:00). Mice were freely assessed to food and water. They were randomly divided into five litters and housed accordingly throughout the behavioral experiment.

### 2.2 Experimental design

Following one week of acclimatization, forty C57BL/6J mice were randomly divided into four groups: Control, CVS + Saline (CVS + Sal), CVS + Fluoxetine (CVS + Flx), and CVS + esketamine (CVS + ek-Ket). Mice in the CVS + Sal, CVS + Flx, and CVS + es-Ket groups were exposed to a 3-week CVS procedure, followed by their respective treatments. Mice in the CVS + Sal and CVS+Flx groups were intraperitoneally injected with either saline (10 µL/g) or fluoxetine (20 mg/kg) once daily for 7 consecutive days. Mice in the CVS + es-Ket group received an intraperitoneal saline injection for 6 days and one esketamine (15 mg/kg) administration on the 7th day. At 24 hours after the last drug administration, depressive and anxiety-like behaviors were evaluated through the open field test, elevated plus maze test, novel suppressed feeding test, tail suspension test, and force swim test. All the mice were sacrificed after those behavioral experiments, and the plasma was collected for later ELISA tests.

### 2.3 Production of depressive and anxiety-like behavior via chronic variable stress procedure

The depressive- and anxiety-like behaviors were established through a CVS procedure reported by previous studies, including ours (15, 24). Briefly, mice were exposed to three different stressors for 21 consecutive days. On day 1, mice were randomly stimulated with 0.45 mA foot shocks 100 times in 1 h. On day 2, mice were tail-suspended for 1 h. On day 3, mice were restrained in a 50 ml tube for 1 h. In all 21 days, mice were repeatedly subjected to the three above stressors with 7 circles.

### 2.4 Drug administration

Fluoxetine (Sigma, St. Louis, MO) was dissolved in distilled water and administrated through *i.p.* injection; the injection volume was 5 mL/kg with the 10mg/kg dosage based on our previous studies. Mice in CVS + Flx and CVS + Sal groups received Flx or saline treatment for 7 consecutive days. Esketamine (25 mg/ml, Hengrui Medicine Co., Ltd) was diluted in saline to a working solution of 1.5 mg/ml. Mice in the CVS + es-Ket group were weighed and treated with saline for 6 consecutive days and a single esketamine administration at day 7 with a 15 mg/kg dosage.

### 2.5 Behavioral assays

As shown in the **supplemental material 1**, all behavioral tests were confirmed 24 h after the last *i.p.* injection of esketamine, fluoxetine, or saline with the following timeline as reported previously: open field test (OFT), novelty suppressed feeding test (NSFT), elevated plus maze (EPM), tail suspension test (TST), and forced swim test (FST) with a 24-hour interval in order to avoid unnecessary stress on rodents (15, 24–26). The mice were acclimated to the test environment for at least 60 minutes before performing behavioral tests. All behavior tests were conducted in the light cycle (8:00 ~ 20:00) under dim lighting conditions, and the test arena was cleaned with 75% ethanol between each trial.

#### 2.5.1 Open field test

Mice were placed in an open field apparatus (50 × 50 × 50 cm) during the OFT analysis, and their movements were recorded through a video track system positioned directly above the box. The Top Scan behavioral analysis system (TopScan, Clever Sys Inc, USA) was used to analyze indicators, including the distance moved and time spent in the center arena, which was over 5 minutes. The floor of the open field box was divided into 36 squares, of which 16 were the central square and 20 were the peripheral square. The distance traveled and time spent in the central arena and the whole squares were analyzed.

#### 2.5.2 Novel suppressed feeding test

In the current study, NSFT was performed using the published protocol, including ours. Briefly, mice were food-restricted for 24 hours before the start of NSFT. During the test, mice were allowed to be habituated in the testing room for 30 min and then placed in an open field box (50 × 50 × 50 cm) covered with bedding. A single food pellet was placed in the center, and the camera positioned directly up the testing arena started to record the whole trial process. The test was stopped once the mouse began eating or when the 10-minute test time ended.

#### 2.5.3 Elevated plus maze test

During the EPM test, mice were gently placed in the center of a plus-shaped apparatus, which consisted of two opposite-open arms (30 cm × 5 cm × 1 cm), two opposite-closed arms (30 cm × 5 cm × 15 cm), and a central zone (5 cm × 5 cm). The plus-shaped apparatus was elevated 50 cm above the ground. During the test, mice were placed in the open arm of the maze facing the center zone. The activity of each free-moving mouse was tracked by a video placed directly above the arena and analyzed by TopScan software. The time spent in the open arms in a total 5-minute test period was recorded to assess the anxiety-like behavior of each mouse.

#### 2.5.4 Tail suspension test

In the TST, mice’s tails were taped to a horizontal pole 50 cm above the floor for 6 minutes, and activity was recorded through a video tracking system. The first minute of the test was considered the mice’s adaptation period. Immobility was defined as loss of skeletal movement for at least 1 second and was analyzed for 5 minutes of the test by an automated tracking software (Freeze Scan, Clever Sys, Inc.). Any mouse that crawled back up its tail was excluded from the data analysis.

#### 2.5.5 Forced swimming test

The FST was confirmed according to our previous report. Briefly, a pre-test was set at 24 hours before the test to allow the mice to habituate the test. During the test, each mouse was singly put in a Plexiglas cylinder with a 14-cm inner diameter and 30-cm height containing water (20 cm of water; 23°C - 25°C) and was allowed to swim freely for 6 min. A video camera was set to record the activity of each mouse throughout the test. The immobility time was calculated with the date in the last 5 min of the test by an automated tracking software (Freeze Scan, Clever Sys, Inc.).

### 2.6 Tissue collection and ELISA

After all behavioral tests were conducted, the mice were euthanized, and the blood was collected on ice immediately for enzyme-linked immunosorbent assay (ELISA) kits (Shanghai FANKEL Industrial Co., Ltd). The blood of mice was collected using heparin anticoagulant tubes containing a small amount of liquid, and the obtained whole blood was thoroughly mixed with the anticoagulant. Plasma samples were obtained by centrifugal force at 3000 rpm for 15 minutes. All samples are stored at -80 °C. The inflammation factors including pro-inflammatory cytokines (IL-1β, IL-6, TNF-α, IL-8, IL-17A, IL-33, and IFN-β, Fankew), anti-inflammatory cytokines (IL-4, IL-9, IL-10, IL-24, IL-37, Fankew) and CXCL12, Arg1 by ELISA kits in according to the specification of manufactures. The reliable standard curve is represented by a linear regression *R* ^2^ value ≥0.95, and the standard range depends on the manufacturer’s instructions.

### 2.7 Statistical analysis

All data in the current study was analyzed using GraphPad Prism 8.0 software. The results were expressed as mean ± standard error (mean ± S.E.). The D’Agostino-Pearson omnibus normality test was used to assess the homogeneity of variance. One-way ANOVA followed by Bonferroni’s *post-hoc* test was applied to compare groups with normally distributed data. While the non-normally distributed data were analyzed using the *Kruskal-Wallis* test and *Dunn’s* post-test. Pearson correlation analysis was used for normally distributed variables. A two-tailed *P*-value < 0.05 was considered with statistically significance (* *P <* 0.05, ** *P <* 0.01 and ***P < 0.001; respectively).

## 3 Results

### 3.1 Both fluoxetine and esketamine elicited anti-depressive- and anxiolytic-like effects in mice exposed to CVS

**Figure 1:**
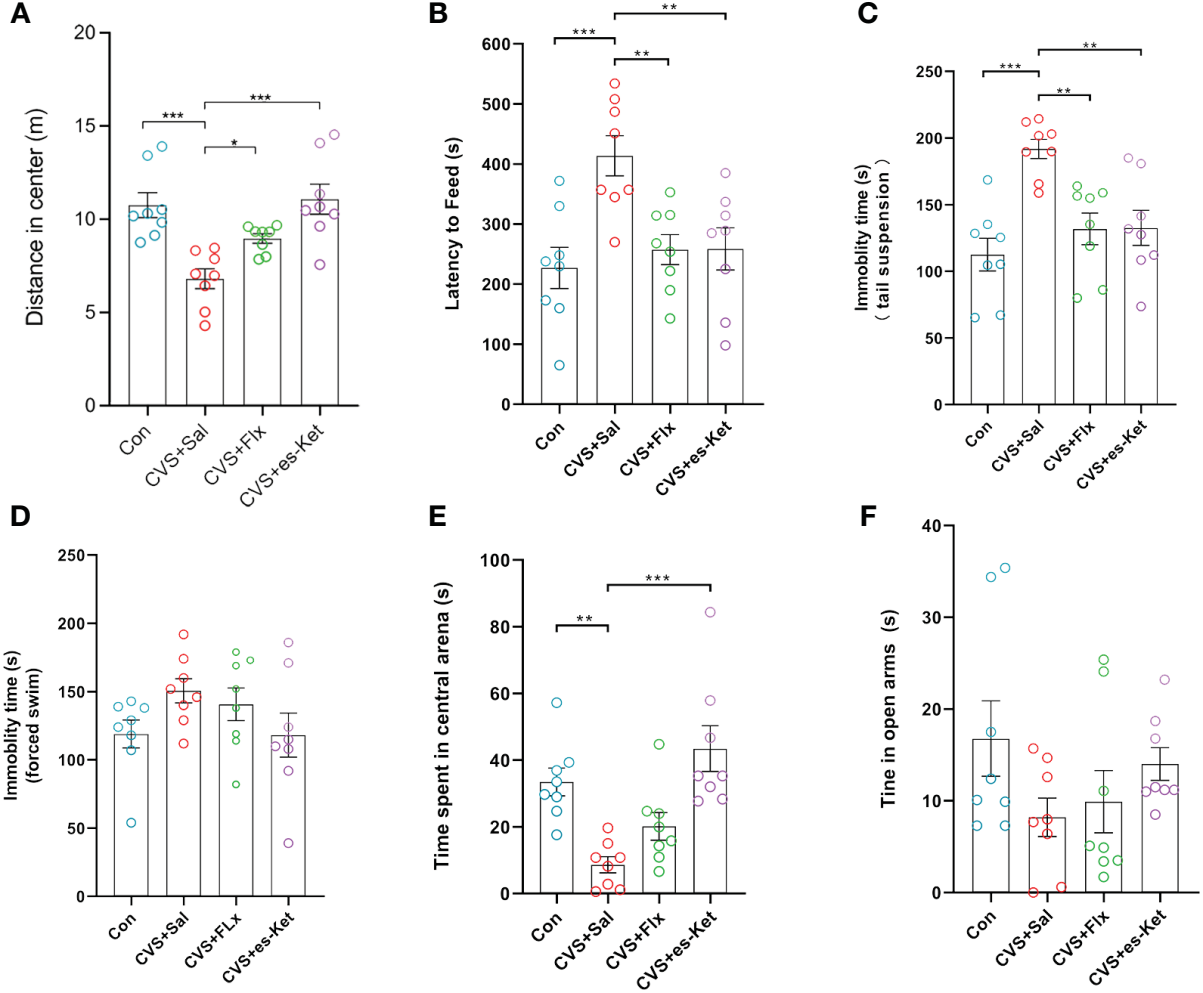
Results of behavioral tests in each group of mice. **(A)** Total distance of open field test; **(B)** feeding latency; **(C)** Tail suspension test standing time; **(D)** Forced swimming immobility time; **(E)** Entry time in the center of the open field; **(F)** Time in open arm. (*); *p*-value ≤ 0.05, (**); *p*-value ≤ 0.01, (***); *p*-value ≤ 0.001.

As illustrated in **Figure 1**, the effects of fluoxetine and esketamine on depressive- and anxiety-like behaviors were investigated. The results indicated that there were significant differences in the total distance moved in the center of OFT (*F_3,28 _= *10.6, *P*=0.01), latency to feed in NSFT (*F_3,28 _= *6.878, *P*=0.001), immobility time in TST and FST(*F_3,28 _= *9.148, *P*=0.001; *F_3,28 _= *1.781, *P*>0.05) among groups; these differences were also detected in anxiety-like behaviors, including time spent in central arena of OFT (*F_3,28 _= *10.06, *P*=0.001), time spent in open arms of EPM(*F_3,28 _= *1.689, *P* = 0.192). No significant differences were detected in the total distance moved in the whole arena (As shown in **supplemental material 2**). The *post hoc* comparisons further indicated that CVS significantly reduced the total distance in the central arena of OFT, which was reversed by fluoxetine or esketamine administration [CVS + saline *vs.* CVS + Fluoxetine, CVS + Saline *vs.* CVS + esketamine; *P <* 0.05, respectively]. Moreover, the significantly increased latency to feeding in NSFT and the immobility time in TST and FST were detected in the CVS + saline group, as compared with the Control group [*P <* 0.05, respectively]. No significant differences among groups were detected in latency to food in the home cage (As shown in **supplemental material 3**). This increased latency to food and the immobility time in TST and FST produced by the CVS procedure were significantly reversed by chronic administration of fluoxetine or acute administration of esketamine [CVS + saline *vs.* CVS + Fluoxetine, CVS + Saline *vs.* CVS + esketamine; *P <* 0.05, respectively]. Moreover, anxiety-like behaviors, including time spent in the central arena of the OFT and in the open arms of the elevated plus maze (EPM), were also shown in **Figure 1**. CVS reduced time spent in the central arena in OFT (*F_3,28 _= *10.06, *P*=0.001) and also decreased time spent in open arms in EPM (*F_3,28 _= *1.689, *P* = 0.192). In contrast, the 7-day administration of fluoxetine or a single injection of esketamine reversed these anxiety-like behaviors [CVS + saline *vs.* CVS + Fluoxetine, CVS + Saline *vs.* CVS + esketamine; *P <* 0.05, respectively].

### 3.2 Both fluoxetine and esketamine attenuated the elevated expression of plasma pro-inflammatory cytokines produced by the CVS procedure

**Figure 2:**
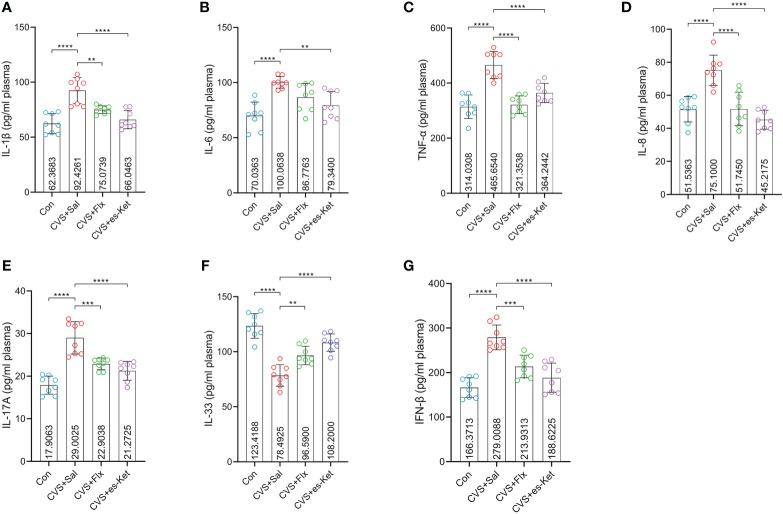
Effects of fluoxetine and esketamine on the expression of pro-inflammatory factors. **(A)** IL-1β; **(B)** IL-6; **(C)**TNF-α; **(D)** IL-8; **(E)** IL-17A; **(F)** IL-33;**(G)** IFN-β (**); p-value ≤ 0.01, (***); p-value ≤ 0.001, (****) p-value ≤ 0.0001.

As shown in **Figure 2**, the effect of fluoxetine and esketamine on the expression of the pro-inflammatory cytokines was first examined. The results of one-way ANOVA analysis indicated there were significant differences in the levels of IL-1β, IL-6, TNF-α, IL-8, IL-17A, IFN-β and IL-33 in plasma(*F_3,28 _= *18.81, *P*=0.001; *F_3,28 _= *10.56, *P*=0.001; *F_3,28 _= *24.09, *P*=0.001; *F_3,28 _= *19.78, *P*=0.001; *F_3,28 _= *27, *P*=0.001; *F_3,28 _= *25.54, *P*=0.001; *F_3,28 _= *32.58, *P*=0.001; respectively). The *post hoc* comparisons further indicated that CVS increased the expression of IL-1β, IL-6, TNF-α, IL-8, IL-17A, and IFN-β (**Figures 2A-E, G**, Control vs. CVS + saline, *P <* 0.0001, respectively) but decreased the level of IL-33 in plasma as compared with those in the control group (**Figure 2F**, Control vs. CVS + saline, *P <* 0.0001). Whereas 7-days fluoxetine treatment reduced the level of IL-1β (CVS + saline vs. CVS + fluoxetine, *P <* 0.01), TNF-α (CVS + saline vs. CVS + fluoxetine, *P <* 0.001), IL-8 (CVS + saline vs. CVS + fluoxetine, *P <* 0.001), IL-17A (CVS + saline vs. CVS + fluoxetine, *P <* 0.001) and IFN-β (CVS + saline vs. CVS + fluoxetine, *P <* 0.001), increased the level of IL-33 (CVS + saline vs. CVS + fluoxetine, *P <* 0.01) in plasma as compared with those in CVS group. No significant difference in the expression of IL-6 was detected between CVS + saline and CVS + fluoxetine groups. Also, as shown in **Figure 2**, the single administration of esketamine reversed the increased expression of IL-1β, IL-6, TNF-α, IL-8, IL-17A IL-33 and IFN-β in plasma (**Figures 2A-E, G**, CVS + saline vs. CVS + es-Ket; *P <* 0.0001, *P <* 0.01, *P <* 0.0001, *P <* 0.0001, *P <* 0.0001, *P <* 0.0001, *P <* 0.0001; respectively) as compared with those in CVS + saline group. As expected, non-significant differences were detected in pro-inflammatory cytokines between CVS + Flx and CVS + es-Ket groups.

### 3.3 Fluoxetine and esketamine administration improve the level of anti-inflammatory cytokines in the plasma of CVS-exposed mice

**Figure 3:**
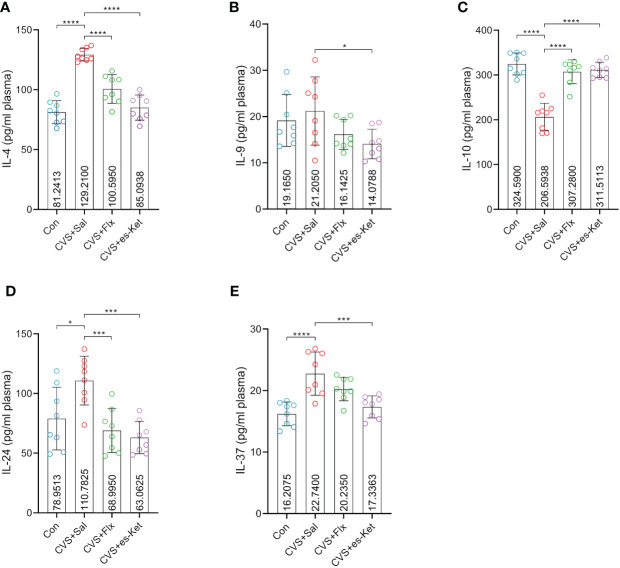
Effects of fluoxetine and esketamine on the expression of anti-inflammatory factors. **(A)** IL-4; **(B)** IL-9; **(C)** IL-10; **(D)** IL-24; **(E)** IL-37. (*); p-value ≤0.05, (***); p-value ≤ 0.001, (****); p-value ≤ 0.0001.

Fluoxetine and esketamine treatments also increased the level of anti-inflammatory cytokines in CVS-exposed mice (**Figure 3**). The results indicated that there were significant differences in the levels of IL-4, IL-10, IL-24, IL-37 and IL-9 in plasma (*F_3,28 _= *40.43, *P*=0.001; *F_3,28 _= *37.8, *P*=0.001; *F_3,28 _= *8.871, *P*=0.0003; *F_3,28 _= *12.12, *P*=0.0001; *F_3,28 _= *2.994, *P*=0.0476; respectively). The *post hoc* comparisons further indicated that the exposure to CVS increased the levels of anti-inflammatory cytokines, including IL-4, IL-10, IL-24, and IL-37 in the mice(Control vs. CVS + Sal, *P <* 0.0001, *P <* 0.0001, *P <* 0.05, *P <* 0.0001; respectively). However, no significant difference in IL-9 between the control and CVS + Sal groups was detected. As compared with mice in the CVS + saline group, mice treated with fluoxetine reversed the increased expression of IL-4, IL-10, and IL-24 (CVS + saline vs. CVS + fluoxetine; *P <* 0.0001, *P <* 0.0001, *P <* 0.01; respectively). At the same time, no significant differences in IL-9 or IL-37 between CVS + saline and CVS + fluoxetine groups were detected. Moreover, the administration of esketamine reduced the increased level of plasma IL-4, IL-9, IL-10, IL-24, and IL-37 produced by the exposure to CVS procedure (CVS + saline vs. CVS + es-Ket; *P <* 0.0001, *P <* 0.05, *P <* 0.0001, *P <* 0.0001, *P <* 0.001, *P <* 0.001; respectively).

### 3.4 The supplementation of fluoxetine and esketamine reversed the increased expression of CXCL12 but not Arg1 induced by the CVS procedure

**Figure 4:**
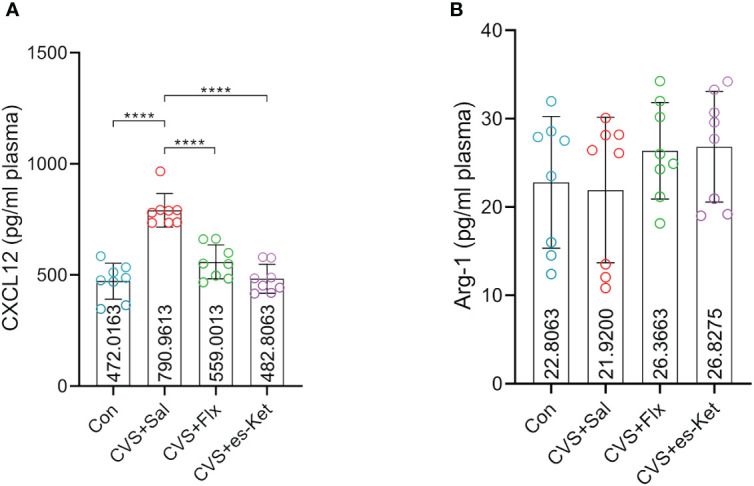
Effects of fluoxetine and esketamine on the expression of CXCL12 and Arg-1 **(A)** CXCL12; **(B)** Arg-1. (*); *p*-value ≤ 0.05, (**); *p*-value ≤ 0.01, (***); *p*-value ≤ 0.001.

**Figure 4** shows the macrophage-related biomarkers, including CXCL12 and Arg1. The results indicated that there were significant differences in the levels of CXCL12 in plasma (F3,28 = 31.53, P=0.001), while there was no significant difference in the levels of Arg1 (*F_3,28 _= *1.022, *P*=0.3977). The *post hoc* comparisons further indicated that the exposure of CVS significantly increased the expression of CXCL12 (Control vs. CVS + Sal, *P <* 0.0001) but not Arg1, compared to the control group. Both fluoxetine and esketamine reduced the elevated plasma level of CXCL12 (CVS + saline vs. CVS + Flx, CVS + saline vs. CVS + es-Ket; *P <* 0.0001), but not Arg1 as compared to that in CVS + Saline group.

### 3.5 Correlation between pro-inflammatory/anti-inflammatory cytokines and depressive and anxiety-like behaviors

**Figure 5:**
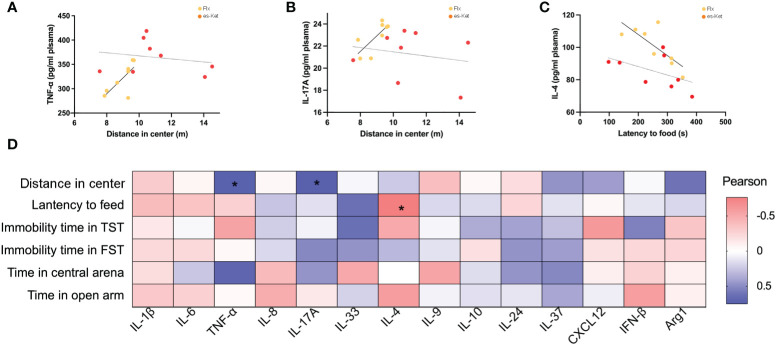
Correlations between inflammatory factor and behaviors in plasma in CVS + Flx. **(A)** TNF-α and total distance in OFT in CVS + Flx/es-Ket; **(B)**IL-17A and total distance in OFT in CVS + Flx/es-Ket; **(C)** IL-4 and latency to food in NSFT in CVS + Flx/es-Ket; **(D)** Pearson correlation heatmap in CVS + Flx. (*); p-value ≤ 0.05.

**Figure 6:**
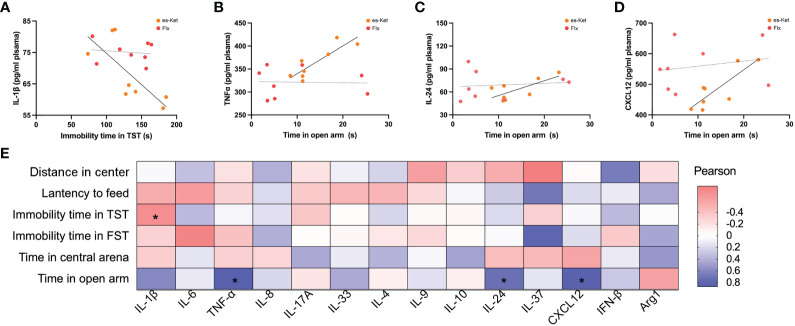
Correlations between inflammatory factor and behaviors in plasma in CVS + es-Ket. **(A)** IL-1β and immobility in TST in CVS + es-Ket/Flx; **(B)** TNF-α and time in open arms in EPM in CVS + es-Ket/Flx; **(C)** IL-24 and time in open arms in EPM in CVS + es-Ket/Flx; **(D)** CXCL12 and time in open arms in EPM in CVS + es-Ket/Flx; **(E)** Pearson correlation heatmap in CVS + es-Ket. (*); p-value ≤ 0.05.

To further elucidate the potential roles of the inflammatory factors on depression and anxiety, a correlation analysis was also performed to assess the representative values of behavioral outcomes and alterations in inflammatory factors (**Figures 5**, **6**). Notably, positive correlations of TNF-α and IL-17A with total distance in the OFT were observed in the fluoxetine treatment group (r=0.7320, *P=*0.0390; r=0.7464, *P*=0.0334; respectively, **Figures 5A, B**). Additionally, IL-4 exhibited a negative correlation with latency to food (r=-0.7650, *P=*0.0270, **Figure 5C**). However, other inflammatory factors had no significant correlation with depressive or anxiety-like behaviors, as shown in **Figure 5D**. In the esketamine treatment group, there was a negative correlation of the expression of IL-1β with immobility time in the TST (r=0.7462, *P=*0.0335, **Figure 6A**). At the same time, a positive relevance was detected in TNF-α, IL-24, and CXCL12 levels and the time spent in open arms in the EPM (r=0.8669, *P=*0.0053; r=0.7139, *P*=0.0467; r=0.8316, *P=*0.0305; respectively, **Figures 6B-D**). Additionally, a negative correlation between the level of IL-1β and immobility time spent in TST was also detected, as shown in **Figure 6E** (*P <* 0.05).

## 4 Discussion

In the current study, both a single dose of esketamine and repeated treatment of fluoxetine elicited anti-depressive and anxiolytic effects and partly normalized CVS-elevated inflammatory response, suggesting that the modulation of plasma inflammatory cytokines might have participated in the anti-depressive and anxiolytic effect of esketamine and fluoxetine. These findings revealed that the plasma inflammatory cytokines could potentially be relevant in the treatment response of anti-depressants.

The CVS model is widely accepted as a model to mimic depressive-related symptoms in human beings induced by prolonged chronic stress. The behavioral, macrobiotic, neural, and inflammatory changes induced by this model closely mirror the characterization in patients with MDD, which can be reversed by anti-depressant treatments such as fluoxetine (24, 25;. In the current study, mice exposed to CVS exhibited depressive-like behaviors such as reduced locomotion in OFT, increased immobility in the tail suspension, and forced swimming tests, which have been reported in our previous study (15). In recent years, some studies, including ours, also reported that a single dose of esketamine could produce rapid anti-depressant effects. Consistent with these reports, we also found that a single dose of esketamine made a rapid antidepressant-like effect similar to repeated supplementation of fluoxetine in various behavioral tests, including OFT, TST, and FST. Additionally, the results regarding time spent in the center of an open field and the open arms of an elevated plus maze indicated that both esketamine and fluoxetine treatment elicited anxiolytic effects in the CVS model. These findings indicated that CVS successfully produced depressive and anxiety-like behaviors in mice, and the treatment of acute esketamine and chronic fluoxetine elicited antidepressant and anxiolytic effects. However, the mechanisms underlying these anti-depressant and anxiolytic actions are still not fully understood.

Compelling studies demonstrated that esketamine exerts its rapid anti-depressant effects through up-regulated glutaminergic transmission, increased synaptogenesis and neurotrophic factor activity (27–30). Additionally, the administration of esketamine has been shown to regulate BDNF/TrkB signaling and reduce NF-κB activation in PFC, thereby reducing pro-inflammatory response and rescuing depressive-like behavior (31). Moreover, peripheral immune dysregulation is also implicated in the pathogenesis of MDD, with inflammatory cytokines emerging as potential biomarkers for disease severity and treatment efficacy. Although peripheral inflammatory cytokines have also emerged as promising candidate biomarkers for the clinical severity and treatment efficacy of MDD, the details of how esketamine and fluoxetine, two anti-depressants with generally different working mechanisms, affected peripheral inflammatory response are still unclear (32–34).

In recent decades, the expression of pro-inflammatory factors, including interleukin-1β (IL-1β), tumor necrosis factor α (TNF-α), interleukin-6 (IL-6), and IL-17A in brain and plasma have been reported in relation with the efficacy of anti-depressants treatment (35, 36). Inconsistent with these studies, our results showed increased plasma levels of pro-inflammatory cytokine IL-1β, IL-6, TNF-α, IL-8, and IL-17A in CVS-exposed mice, which was reversed by the supplementation with esketamine. Moreover, no significant difference was detected between esketamine and fluoxetine in the current study in these pro-inflammatory cytokines. These findings indicated that a single dose of esketamine and repeated fluoxetine treatment reduced the elevated expression of these pro-inflammatory cytokines induced by CVS. However, only esketamine but not fluoxetine reversed the overexpressed expression of IL-6; this indicated that esketamine might regulate the plasma pro-inflammatory cytokines in a different mechanism than fluoxetine. Some recent studies reported that ketamine and fluoxetine elicited their antidepressant capacity through different underlying mechanisms, including mitochondrial function, but more details still need to be supplied. Moreover, the current study also examined the plasma level of IL-33, a novel pro-inflammatory cytokine with multiple functions, and found that mice exposed to CVS exhibited a significant decrease in IL-33, which aligns with recent studies (37, 38). The administration of esketamine and fluoxetine treatment reversed the increased expression of IL-33. These results indicated that the rapid and slow-onset antidepressants altered the expression of pro-inflammatory cytokines.

Besides pro-inflammatory cytokines, several studies found that the expression of some anti-inflammatory cytokines, including IL-4 and IL-10 was lower in MDD patients than those in healthy individuals (39, 40). Our study found a significant decrease of IL-10 but not IL-4 levels in the plasma of mice exposed to CVS. This indicated that there was a difference in anti-inflammatory response between human beings and rodents. In the current study, we also tested levels of IL-24 and IL-37, two anti-inflammatory cytokines linked to various immunopathological-related diseases. The increased IL-24 haplotype has been found to be associated with an increased risk of MDD. Additionally, IL-37 was reported to be involved in regulating inflammatory processes in relevance to depression. Our study found that exposure to CVS led to a significant decrease in plasma levels of IL-4, IL-10, IL-24, and IL-37 in mice. The administration of fluoxetine increased the expression of IL-4, IL-10, and IL-24. Meanwhile, esketamine restored the plasma anti-inflammatory cytokines IL-4, IL-10, IL-24, and IL-37 in plasma. There were slightly different underlying mechanisms in the regulation of anti-inflammatory cytokine between these two different types of antidepressants.

Increasing evidence indicated that the macrophage-related chemokine system is the main protein responsible for regulating inflammatory processes in the brain via peripheral-central crosstalk. The pro-inflammatory cytokines and chemokines, for instance, interleukin-8 (IL-8) or C-X-C motif chemokine ligand 8 (CXCL8) produced by macrophage, microglia, or endothelial cells (41). Recently, some studies have proposed a role for these chemokines in the development of mental disorders, including MDD. It has been reported that higher levels of IL-8 in the cerebrospinal fluid (CSF) were about the severity of bipolar and unipolar depression in elderly patients (42, 43). However, a randomized study using whole blood samples reported that patients with higher levels of IL-8 were less likely to develop inflammation-related depression (44). The current study also examined the changes in plasma of IL-8 (CXCL8), CXCL12, and another macrophage-related cytokine, Arg1, in mice exposed to CVS. Our results showed an increase in IL-8 in CVS-exposed mice, further supporting the link between IL-8 and depression. CXCL12 synergistically acted in inflammatory conditions with CXCL8, which exerted a pro-inflammatory response (41). Multiple studies have found that plasma concentrations of CXCL-12 are higher in patients with MDD than in healthy control individuals (45, 46). A preclinical study on the prenatal stress-induced depressive-like model found the stress-evoked CXCL-12 upregulation in the hippocampus and frontal cortex, while the chronic fluoxetine treatment normalized the CXCL12-CXCR4-CXCR7 axis (47). Our results showed that CVS exposure significantly increased plasma CXCL12 levels, which was reversed by fluoxetine and esketamine treatment. This indicated that the rescued peripheral chemokines level correlates with the anti-depressant effect of esketamine, which is consistent with previous findings.

A correlation analysis was also conducted in the current study to explore further the correlation between depressive and anxiety-like behaviors and changes in plasma inflammatory mediators. The results showed that peripheral plasma levels of IL-1β, IL-6, TNF-α, IL-17A, IL-4, IL-24, IL-37, IL-8, CXCL12, and INF-β were positively correlated, while the level of IL-10 was negatively correlated with depressive-like behaviors. Peripheral plasma levels of IL-1β, IL-6, TNF-α, IL-4, and INF-β were negatively correlated, while IL-33, IL-9, and Agr1 positively correlated with anxiety-like impairments. The levels of IL-17A, IL-10, IL-24, IL-37, and CXCL12 were only negatively correlated with time in the center in OFT, which is used to evaluate the spontaneous activity ability of mice. In addition, the level of IL-33 was negatively correlated with immobility time in TST while positively correlated with feeding latency in NSFT. This result suggested that we need to refine further and classify behavioral indicators to provide a basis for subsequent mechanism research.

## 5 Limitations

While our study provides valuable insights into the effects of fluoxetine and esketamine on plasma inflammatory cytokines and their role in depression and anxiety-like behaviors, some limitations should be acknowledged. Firstly, the current study was limited to examining plasma inflammatory cytokines in response to anti-depressant treatment. Some studies have revealed the different impacts of esketamine and fluoxetine on neuronal firing mitochondrial function. However, future studies should investigate whether fluoxetine and esketamine exert their anti-depressant effects through similar or different signaling pathways, including the modulation of central inflammatory responses (6, 7, 48).

Secondly, as the natural mediators of the brain’s inflammatory response, microglia also play a direct role in the pathogenesis of depression (49). However, we did not clarify the specific mechanisms of the anti-depressant effect of esketamine versus fluoxetine in regulating microglia phenotype or function. Whether these complex microglia phenotypes are directly or indirectly affected by esketamine and whether this phenotypic polarization is critical to the mechanism of esketamine’s rapid anti-depressant action that distinguishes it from traditional anti-depressants remains to be determined.

## 6 Conclusions

In conclusion, our study demonstrates that a single administration of esketamine exerts rapid anti-depressant and anxiolytic effects by modulating levels of peripheral inflammatory mediators to limit damage from CVS exposure-induced peripheral inflammation. This study suggested that the mechanism may be related to phenotypic transformation and functional changes in microglia. Our studies indicate that esketamine may offer a promising treatment option for mood disorders associated with peripheral inflammation. Future research should further elucidate the mechanisms underlying the anti-depressant effects of esketamine and explore its clinical potential in patients with depression and anxiety disorders.

## Data availability statement

The original contributions presented in the study are included in the article/supplementary material. Further inquiries can be directed to the corresponding authors.

## Ethics statement

The animal study was approved by The Institutional Animal Care and Use Committee of the Fourth Military Medical University. The study was conducted in accordance with the local legislation and institutional requirements.

## Author contributions

HC: Investigation, Methodology, Writing – original draft. XZ: Formal analysis, Investigation, Methodology, Writing – original draft. XM: Data curation, Formal analysis, Investigation, Methodology, Writing – original draft. HM: Data curation, Investigation, Methodology, Writing – original draft. CZ: Resources, Supervision, Writing – review & editing, Funding acquisition. YZ: Formal analysis, Methodology, Writing – review & editing. ZP: Validation, Writing – review & editing. SX: Funding acquisition, Project administration, Supervision, Writing – review & editing. MC: Funding acquisition, Methodology, Supervision, Writing – review & editing.
