# Supplementary material for: Effects of esketamine and fluoxetine on depression-like behaviors in chronic variable stress: a role of plasma inflammatory factors
Source: Front Psychiatry. 2024 May 15;15:1388946. doi: 10.3389/fpsyt.2024.1388946 (PMC11133692; doi:10.3389/fpsyt.2024.1388946)
Supplement: Supplementary file 1 [file DataSheet_1.docx]

**Supplemental material 1 Timeline for the experimental design in the current study**

**
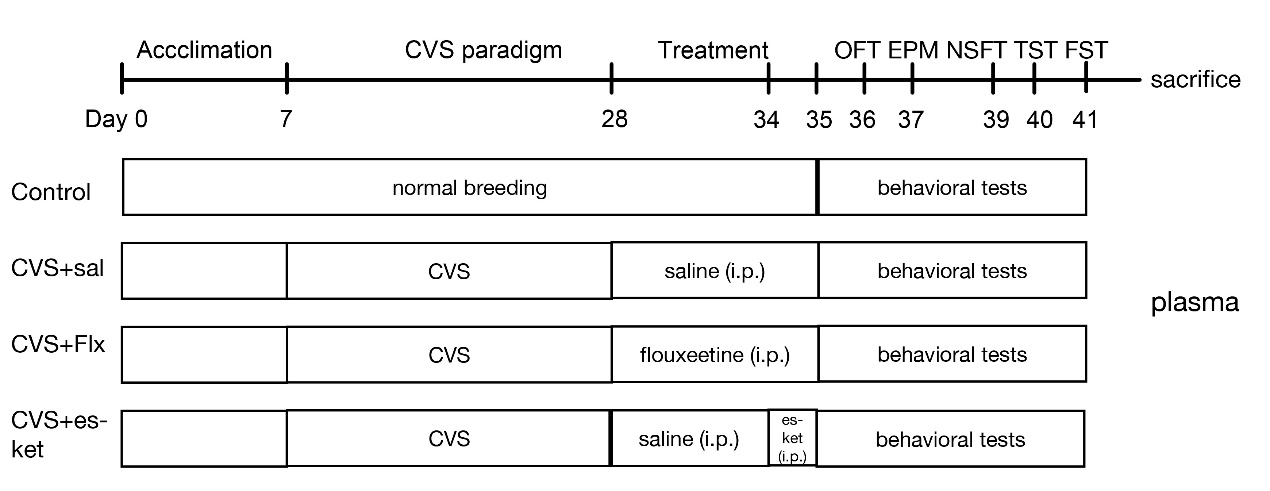
**

**Supplemental material 2 Total distance in the whole area of OFT among groups**


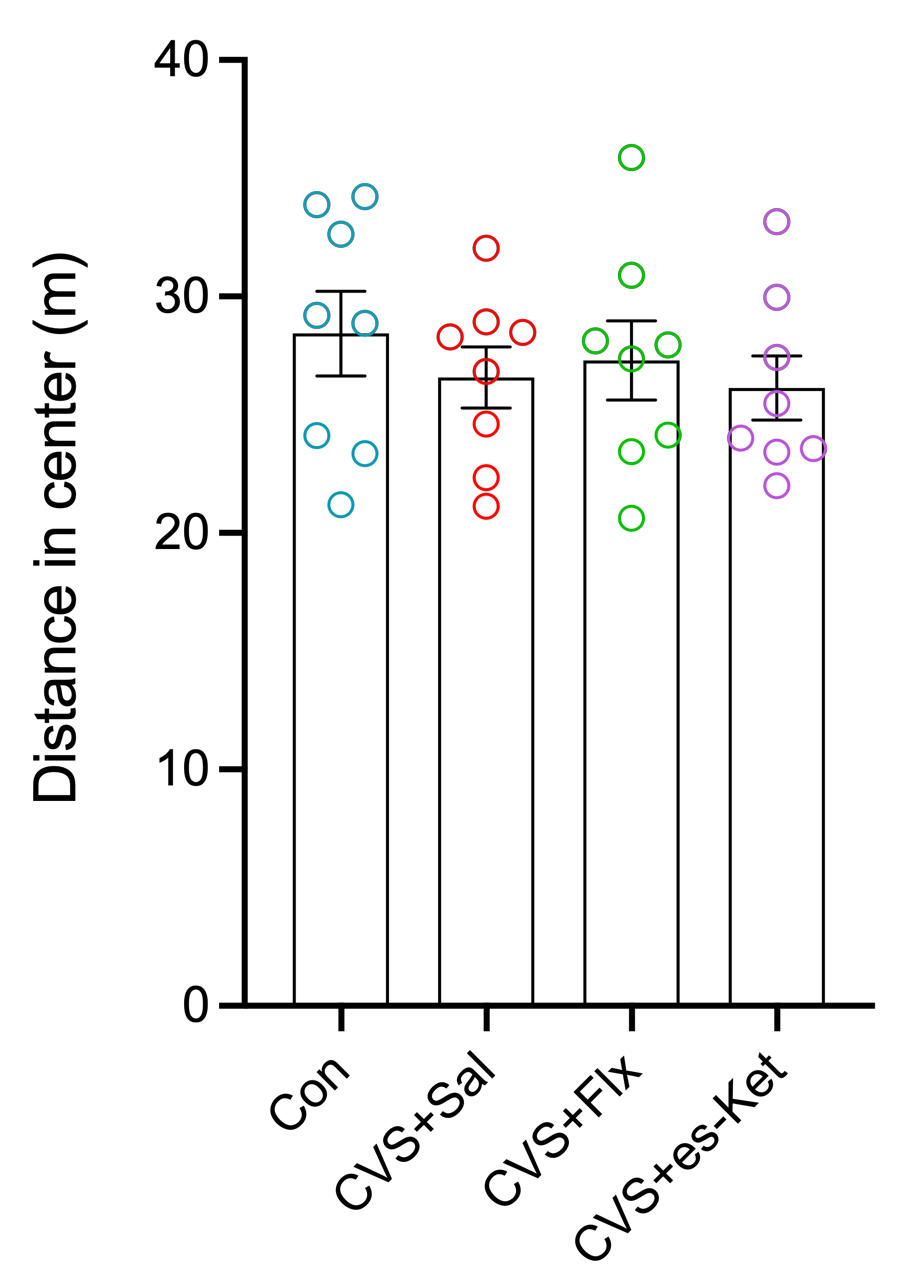


|  | Con | CVS+Sal | CVS+Flx | CVS+es-Ket |
| --- | --- | --- | --- | --- |
| Mean | 28.44 | 26.58 | 27.30 | 26.14 |
| Std. Error of Mean | 1.787 | 1.292 | 1.673 | 1.349 |

**Supplemental material 3 Latency to food in the home cage among groups.**

|  | Con | CVS+Sal | CVS+Flx | CVS+es-Ket |
| --- | --- | --- | --- | --- |
| Mean | 83.93 | 79.18 | 83.63 | 81.16 |
| Std. Error of Mean | 5.013 | 4.928 | 4.847 | 4.732 |
